# Supplementary material for: Water and Nutrient Recovery for Cucumber Hydroponic Cultivation in Simultaneous Biological Treatment of Urine and Grey Water
Source: Plants (Basel). 2023 Mar 12;12(6):1286. doi: 10.3390/plants12061286 (PMC10053017; doi:10.3390/plants12061286)
Supplement: Supplementary file 1 [file plants-12-01286-s001.zip › plants-2255850-supplementary.pdf]

Supplementary materials

Water and Nutrient Recovery for Cucumber Hydroponic Cultivation in Simultaneous Biological Treatment of Urine and Grey Water

Anna Wdowikowska, Małgorzata Reda, Katarzyna Kabała, Piotr Chohura, Anna Jurga, Kamil Janiak and Małgorzata Janicka

Table 1S. Macro- and microelement composition of NUG-cultivated cucumber plants, content in whole plants (n = 5). All parameters in the analyzed variant (NUG) differed from those obtained in reference (RCF solution) in a statistically significant way ($p \leq 0.05$).

Parameters in g·kg DM⁻¹					
N	P	K	Ca	Mg	S
11.2 ±0.49	2.3±0.12	11.2±0.1	8.4±0.24	0.8±0.05	1.1±0.05
**Parameters in mg·kg DM⁻¹**					
Fe	Mn	Cu	Zn	B	Na
5.29±0.06	9.25±0.05	0.00	8.6±0.08	0.00	1450±3.6

Table 2S. Descriptions of analyzed parameters of chl *a* fluorescence transient OJIP emitted by dark-adapted cucumber leaves.

Fluorescence parameter	Description
T for Fm	Time to reaching of Fm
Area	Area above the fluorescence curve between Fo and Fm proportional to the pool size of the electron acceptors Q_A on the reducing side of PSII
Fo	Fluorescence origin, minimal fluorescence of chl *a* in dark adapted leaves, all Q_A electron acceptors in PSII reaction centers (RC) are fully oxidized and ready to absorption of light pulse
Fm	Maximal fluorescence of chl *a*, all Q_A electron acceptors in PSII are fully reduced with saturating light pulse
Fv	Variable fluorescence, relates to the maximum capacity for photochemical quenching, the result of subtracting Fo from Fm value
Fo/Fm	The efficiency of a water-splitting complex
Fv/Fm	Maximum quantum efficiency of PSII, indicates capacity of PSII for photochemical quenching of energy within PSII
ABS/RC	Absorption of photon flux of antenna chl *a* per reaction center (RC)
Tro/RC	Trapping flux by active PSII units leading to Q_A reduction per RC
Dio/CSo	Dissipated energy flux in PSII antenna in process other than trapping per cross section
Eto/RC	Energy flux of electron transport from Q_A to further electron acceptors per RC
PI_{ABS}	Performance index for energy conservation from excitation on to the reduction of PSI end acceptors, indicator of sample vitality

Table 3S. Significance of selected monitored parameters for plant physiology.

Monitored parameter	Significance for plants
H_2O_2	one of the reactive oxygen species
lipid peroxidation	indicator of the degree of oxidative lipid degradation by free radicals
CAT	component of the cell antioxidant system;<br>H_2O_2-scavenging enzyme responsible for the conversion of hydrogen peroxide to water and molecular oxygen
APX	component of the cell antioxidant system;<br>H_2O_2-scavenging enzyme responsible for the reduction of H_2O_2 to water using ascorbate as a specific electron donor
proline	a proteogenic amino acid;<br>a compatible solute involved in the amelioration of oxidative stress
NR	enzyme that catalyses reduction of nitrate to nitrite, first reaction of assimilation of inorganic nitrogen into organic compounds
chlorophyll a, chlorophyll b, carotenoides	pigments involved in photosynthesis light reactions, which absorb photon energy

Section 1S. Urine and grey water nitrification procedure.

The production of the fertilizer was conducted with the use of the nitrification process in a 150 L pilot scale Sequencing Batch Reactor (SBR), which is the same as that used in our previous study for urine nitrification (Janiak et al., 2021) [39]. For the start-up, the reactor was seeded with activated sludge from a wastewater treatment plant and then diluted with technological water. All major processes, such as filling; decanting; the control of pH, oxygen, and temperature; as well as mixing and aeration were maintained automatically using real time data acquisition. The control of temperature was maintained with an internal heater (ETG-K D50 AISI 316L, Termik, 1.5 kW), and with SBR thermal insulation that reduces heat loss. The set temperature was 30 ± 0.5 °C. The process was conducted in 5 cycles per day. Each cycle lasted 288 min, and included dosing (5 min), aeration and mixing (238 min), sedimentation (40 min), and decantation (5 min). The SBR was aerated and stirred with the use of a 60W ES/ET 105 air pump (Charles Austen Pumps Ltd) and a 370 W HM-191 stirrer (Kacperek). A TH25 effluent and influent peristaltic pump with a maximum flow of 1900 mL min^{-1} (18W and 50W) was used for the decantation and raw stream dosing. No excess sludge removal was conducted, and therefore the sludge retention time was more than 100 days.

The oxygen concentration was maintained at a set point level of 3.5 $gO_2{\cdot}m^{-3}$. The alkalinity and pH correction were conducted automatically with the use of 8% $NaHCO_3$. pH was maintained in the range of 7.0–5.9 at various stages of the reactor's operation. Nitrified stream was collected in a 50 L tank, where the collection of samples was conducted for periodic measurements of effluent quality.

References:

39. Janiak, K.; Jurga, A.; Wizimirska, A.; Miodoński, S.; Muszyński-Huhajło, M.; Ratkiewicz, K.; Zięba, B. Urine Nitrification Robustness for Application in Space: Effect of High Salinity and the Response to Extreme Free Ammonia Concentrations. J Environ Manage 2021, 279, doi:10.1016/j.jenvman.2020.111610.
